# Postoperative differences between colonization and infection after pediatric cardiac surgery-a propensity matched analysis

**DOI:** 10.1186/1749-8090-8-166

**Published:** 2013-07-02

**Authors:** Daniel J Lex, Roland Tóth, Zsuzsanna Cserép, Tamás Breuer, Erzsébet Sápi, András Szatmári, János Gál, Andrea Székely

**Affiliations:** 1 School of PhD Studies, Semmelweis University, Üllői út 26, 1085 Budapest, Hungary; 2 Department of Anesthesia and Intensive Care, Gottsegen György Hungarian Institute of Cardiology, Haller u. 29, 1096 Budapest, Hungary; 3 Department of Pediatric Cardiology, Gottsegen György Hungarian Institute of Cardiology, Haller u. 29, 1096 Budapest, Hungary; 4 Department of Anesthesiology and Intensive Therapy, Semmelweis University, Kútvölgyi út 4, 1125 Budapest, Hungary

**Keywords:** Colonization, Infection, Conversion, Risk factors, Pediatric, Cardiac surgery, Perioperative, Intensive care

## Abstract

**Background:**

The objective of this study was to identify the postoperative risk factors associated with the conversion of colonization to postoperative infection in pediatric patients undergoing cardiac surgery.

**Methods:**

Following approval from the Institutional Review Board, patient demographics, co-morbidities, surgery details, transfusion requirements, inotropic infusions, laboratory parameters and positive microbial results were recorded during the hospital stay, and the patients were divided into two groups: patients with clinical signs of infection and patients with only positive cultures but without infection during the postoperative period. Using propensity scores, 141 patients with infection were matched to 141 patients with positive microbial cultures but without signs of infection. Our database consisted of 1665 consecutive pediatric patients who underwent cardiac surgery between January 2004 and December 2008 at a single center. The association between the patient group with infection and the group with colonization was analyzed after propensity score matching of the perioperative variables.

**Results:**

179 patients (9.3%) had infection, and 253 patients (15.2%) had colonization. The occurrence of Gram-positive species was significantly greater in the colonization group (p = 0.004). The C-reactive protein levels on the first and second postoperative days were significantly greater in the infection group (p = 0.02 and p = 0.05, respectively). The sum of all the positive cultures obtained during the postoperative period was greater in the infection group compared to the colonization group (p = 0.02). The length of the intensive care unit stay (p < 0.001) was significantly longer in the infection group compared to the control group.

**Conclusions:**

Based on our results, we uncovered independent relationships between the conversion of colonization to infection regarding positive *S*. *aureus* and bloodstream results, as well as significant differences between the two groups regarding postoperative C-reactive protein levels and white blood cell counts.

## Background

Postoperative infection is one of the most serious complications that occur after cardiac surgery in children; its incidence remains high (13-31%) despite recent advances in surgical and intensive care therapies [1-4]. Surgical site infections (SSI; incidence 2.3-8%), septicemia (incidence 6.3-15%), mediastinitis (incidence 0.2-3.3%), and endocarditis (incidence 0.2%) have previously been used for categorization [5,6].

Postoperative infection is one of the most important and leading causes of increased morbidity, such as greater antibiotic usage, more reoperations, and prolonged hospital and intensive care unit (ICU) stays, thus also augmenting treatment costs and increasing resource utilization [7]. Postoperative infection is also a major contributor to increased mortality [3,4,8-10]. According to the Predisposition, Infection, Response, Organ failure (PIRO) concept, the site of infection (particularly bacteremia) and types of pathogens involved are important factors in the development of infection [11].

Colonization refers to microorganisms (found within the body, wound or artificial devices) that do not cause tissue damage; some even exist in a mutualistic or commensal relationship with the host. The following variables are associated with a microorganism becoming a pathogenic factor: the entry route of the organism and its access to distant regions, the immune status of the host system, the virulence of the particular pathogen and the quantity or load of the inoculant.

Several studies have evaluated the risk factors for postoperative infection and found relationships between longer preoperative and ICU stay, open chest after surgery, cyanotic heart disease, younger age, and higher complexity scores [1,2,4]. Few studies have compared innocuous bacterial colonization with clinically confirmed infection in terms of mortality and outcomes [12]. Furthermore, no study has evaluated a simple scoring system to successfully estimate the patient risk for major infection after cardiac surgery [13].

The purpose of our study was to compare preoperatively non-infected patients with positive bacterial samples with patients who developed infection during the postoperative period and to investigate potential vulnerability factors of microbial colonization associated with the development of postoperative infection. To eliminate the differences arising from the heterogeneity of perioperative factors between the two groups, we used propensity matching analysis.

## Methods

### Patient demographics

Institutional Review Board (TUKEB, Hungary) approval was given for the selected data, which was collated from a prospectively collected database of consecutive pediatric (<18 years) patients undergoing cardiac surgery who were admitted to our cardiac ICU between January 1, 2004 and December 31, 2008; the requirement for parental informed consent was waived. During the study period, 1665 cardiac surgeries were performed. For the patients who underwent more than one operation during the index hospitalization period, only the first operation was considered for the present analysis. Patients were divided into two groups: patients with clinical signs of infection and patients with only positive cultures but without infection during the postoperative period. Because previous studies implicated preoperative nasal colonization as an important risk factor for infection at cardiac sites [14,15], we excluded the patients with bacterial contamination of the anterior naris and throat, infectious endocarditis, and any preoperative infection.

The perioperative data were obtained from a prospectively collected institutional database, which collected data for quality control measurements. The database contained 5 sections that were completed consecutively by a cardiologist (preoperatively and postoperatively), an anesthesiologist, a surgeon, and an intensivist until the patient was discharged. Patient data was matched to the Department of Biochemistry database by using patient identifiers and operation dates. The cardiac surgical procedures were graded as Class 1–6 according to the complexity of the surgery using the Risk Adjustment for Congenital Heart Surgery version 1 (RACHS) method [16]. To quantify the amount of cardiac support, we calculated the modified inotropic score, as described by Wernovsky [17]: [dopamine (μg/kg/min)+dobutamine (μg/kg/min)+100 × epinephrine(μg/kg/min)+100 × norepinephrine (μg/kg/min)+20 × milrinone (μg/kg/min)].

### Outcome assessment

Infection was defined by the pediatric Systemic Inflammatory response Syndrome (SIRS) guidelines [18], which consisted of hyperthermia (≥ 38,5°C), hypothermia (≤ 36°C), and tachycardia (> 2SD according to age), or bradycardia in children aged under 24-months (< 10 percentile), hyperventilation (> 2SD according to age), or the need for mechanical ventilation and white blood cell count (WBC) derangement (elevation or decrease, according to age), or > 10% immature neutrophils in the peripheral blood. Microbiologic confirmation and at least two of the above factors had to have been present, one of which must have been the core temperature or leukocyte change.

Positive microbiological data were examined for each patient, including the date of the first result and the source of the specimen and pathogens found. A total of 4533 positive results were analyzed, which resulted in 22 different types of pathogens being found, including Gram-positive, Gram-negative and fungal microbes from 14 different sources, including wound associated sites, orifices, catheters and other devices.

Mortality was defined as in-hospital death after arriving in the ICU. The combined endpoint of the study was defined as death after ICU arrival (including the patients who died after being transferred to another hospital) or developing multiple organ dysfunction, which consisted of any two of the following complications: 1) postoperative low output syndrome (tachycardia, oliguria, cold extremities or cardiac arrest and an increase in the base deficit of > 4 on two consecutive blood gas measurements), 2) pulmonary complication (defined as non-infectious), 3) nonvascular oxygenation problems (atelectasia, pneumothorax, chylothorax, and phrenic paresis), 4) renal failure (peritoneal dialysis or hemodialysis), and 5) neurological events (convulsions without a prior history or hemorrhage or infarcts demonstrated by cranial imaging), which were also included in the composite outcome. An intensivist and a cardiologist separately determined the patient outcomes that were included in the database during the patient discharge from the hospital.

### Statistical analysis

The data were summarized using descriptive statistics, which were expressed as counts and percentages for the categorical variables and as the means and standard deviation for the continuous data. Patients with missing data regarding their baseline covariates and clinical outcomes were excluded from the analysis. Demographic and perioperative differences between the patients were compared using the chi-squared test, Fisher’s exact test and t-tests, as appropriate.

Because there were differences in the baseline characteristics, the colonization and infection groups were not comparable with respect to important covariates. To minimize the differences and overcome the bias resulting from the study design, we constructed a propensity score model for patients having clinically defined infection or not. The propensity score was constructed using multivariable logistic regression, with infection as the binomial dependent variable and all of the measured covariates that could be related to it (20 variables) as predictor variables. The model’s reliability and predictive ability were measured with the Hosmer-Lemeshow test and the c-index, respectively.

The propensity score derivation model was used to calculate the propensity score for each patient. Based on this score, each patient with infection was matched to a unique control patient with bacterial colonization. A 5→1 computerized greedy matching technique was employed for this matching process, in which cases were first matched to controls that had propensity scores that were identical in all 5 digits. Those cases that did not match were then matched to controls using 4 digits of the propensity score. This process continued down to a 1-digit propensity score match for those that remained unmatched. The measured covariates and outcomes in the matched group were compared between the 2 groups with paired t-test or Wilcoxon signed rank test for continuous variables and McNemar’s test for categorical variables. The procedure yielded 141 well-matched pairs. The 141 matched pairs were analyzed for differences in their baseline characteristics and in the aforementioned predefined outcome variables. The selection of the predictor variables was based on our previous results [19], and a custom Java code generator determined all of the possible variations of the confounders that yielded a standardized difference within a 10% range.

The propensity score derivation model included the following 20 variables: weight, logarithmic transformation of age, cyanosis, univentricular heart, RACHS score, previous operation, preoperative pulmonary hypertension and congestive heart failure, preoperative inotrope treatment and captopril medication, preoperative ICU stay, preoperative mechanical ventilation, cardiopulmonary bypass (CPB) time, cross-clamp time, operation time, minimum nasopharyngeal temperature, deep hypothermic cardiac arrest, ultrafiltration, post-bypass inotropic score, the need for nitric oxide, transfusion (mL/ kg), and the use of aprotinin. The model was reliable (Hosmer- Lemeshow test p = 0.3) and discriminative (c-index = 0.94). Using this model, 141 (of 253) patients with bacterial colonization were matched to 141 (of 179) patients with infection.

All of the tests were two-sided. The SPSS 18.0 statistical software (SPSS Inc., Chicago, IL) was used. A p value of < 0.05 was considered to be statistically significant.

## Results

**Figure 1:**
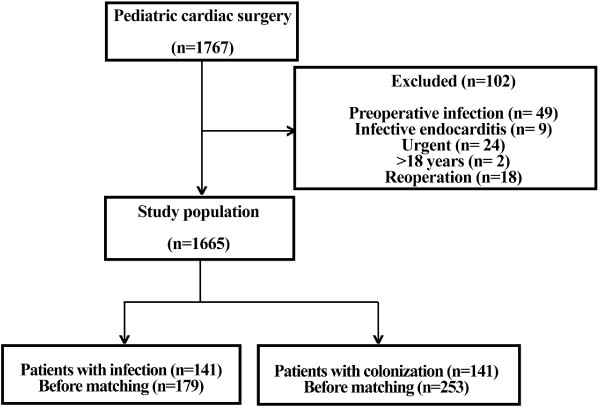
Patient inclusion and allocation.

During the 5-year period, 1665 patients received operations. The analyzed database contained the data of 270 neonates (17.9%), 517 infants (34.2%) and 723 children (47.9%). In the study population of 1665 patients, 179 patients (9.3%) had clinically confirmed infection, and 253 patients (15.2%) had bacterial or fungal colonization without any signs of systemic inflammatory response (Figure 1).

Table 1 shows the comparison of the measured covariates after propensity matching. The mean propensity scores for the colonization and infection groups were 0.367 and 0.377, respectively (p = 0.99). The propensity score of the unmatched infection group was 0.845. Before propensity matching, the patients with infection were younger, had longer hospital stays and required higher doses of inotropics, nitric oxide and red blood cells during surgery; additionally, the occurrence of preoperative pulmonary hypertension was greater. There was a greater occurrence of infection in patients with Down’s syndrome before propensity matching (see Additional file 1: Table S1). After matching the patients, the only remaining difference was increased overall hospital stay in the infection group.

**Table 1:** Demographic and intraoperative variables

	**After propensity matching**				
	**Infection (n=141)**		**Colonization (n=141)**		
**Variable**	**Mean/N**	**SD/%**	**Mean/N**	**SD/%**	**p-value**
**Age (days)**	444.1	967.6	352.1	858.3	0.32
**Gender (male)**	63	45%	61	43%	0.92
**Reoperation**	30	21%	23	16%	0.35
**RACHS (point)**	2.7	1.06	2.7	1.1	0.61
**Preop. cyanosis**	68	48%	71	50%	0.81
**Preop. ICU**	65	46%	68	48%	0.79
**Preop. inotrope**	33	23%	33	23%	0.99
**Preop. captopril**	32	23%	31	22%	0.99
**Pulmonary hypertension**	46	33%	36	26%	0.22
**Down's syndrome**	6	4%	8	6%	0.75
**CPB time (min)**	104.6	86.2	101.3	86.7	0.75
**Cross-clamp time (min)**	49	48.3	43.3	42.1	0.32
**Operation time (min)**	223.7	135.2	215.4	124.8	0.61
**T min nasal (°C)**	31.5	3.9	31.5	4.4	0.94
**DHCA**	4	3%	6	4%	0.75
**Ultrafiltration**	21	15%	17	12%	0.58
**Nitric oxide**	26	18%	27	19%	0.99
**Fluid balance (ml/kg)**	59.4	48.5	52.7	56.5	0.34
**RBC (ml/kg)**	41.4	33.8	39.4	31.1	0.54
**Aprotinin**	33	23%	32	23%	0.99

Data are presented as the number of occurrences and the percentage or as the mean and standard deviation (SD) for categorical and continuous variables, respectively. For categorical variables, the p-values are based on a two-sided chi-squared or Fischer’s exact tests (as appropriate), comparing the infection patients to the colonization patients. For continuous variables, the p-values are based on a t-test or Wilcoxon rank sum test. *RACHS*: risk adjusted congenital heart surgery, *ICU*: intensive care unit; *DHCA*: deep hypothermic cardiac arrest; *RBC*: red blood cells; *CPB*: cardiopulmonary bypass.

Table 2 shows the laboratory results of the patients in each group. The C-reactive protein (CRP) levels on the first and second postoperative days were significantly greater in the infection group (p = 0.02 and p = 0.05, respectively). The leukocyte count and the urea nitrogen (BUN) levels on the first postoperative day were greater in the infection group (p = 0.02 and p = 0.03, respectively).

**Table 2:** Laboratory results

	**After propensity matching**				
	**Infection (n=141)**		**Colonization (n=141)**		
**Variable**	**Mean**	**SD**	**Mean**	**SD**	**p-value**
**Blood sugar DOS max (mmol/l)**	9.1	3.3	8.7	3.1	0.33
**mean (mmol/l)**	6.4	1.7	6.4	1.8	0.97
**min (mmol/l)**	3.9	1	4.4	2.4	0.39
**Blood sugar day 1 max (mmol/l)**	8.5	2.7	8.2	2.8	0.51
**max (mmol/l)**	6.4	1.5	6.2	1.4	0.25
**Blood sugar day 2 max (mmol/l)**	7.4	1.9	7.2	2.2	0.39
**mean (mmol/l)**	5.9	1.4	5.8	1.5	0.56
**Base excess DOS (mEq/l)**	−5.5	3	−6.2	3.4	0.06
**Base excess day 1 (mEq/l)**	−4.1	3.1	−3.7	3.1	0.36
**Base excess day 2 (mEq/l)**	−2.1	3.2	−1.5	3.4	0.22
**CRP day 1 (IU)**	49.6	32.4	39.3	26.8	**0.01**
**CRP day 2 (IU)**	84.9	50.2	72.6	42	**0.05**
**CCl preoperative (mL/min/1.73m**^**2**^**)**	61.7	27.7	56.3	31.7	0.15
**CCl day 1 (mL/min/1.73m**^**2**^**)**	50.7	21.8	50.4	28.5	0.92
**CCl day 2 (mL/min/1.73m**^**2**^**)**	46.7	25	48.9	34.7	0.64
**WBC day 1 (G/l)**	13.1	5	11.9	4.1	**0.02**
**WBC day 2 (G/l)**	13.1	4.9	13.5	4.9	0.65
**Albumin day 1 (g/l)**	26.2	5.5	26.8	6.7	0.51
**Albumin day 2 (g/l)**	28.9	5.1	29.6	6	0.46
**BUN day 1 (mmol/l)**	5.9	3.3	5.1	2.4	**0.03**
**BUN day 2 (mmol/l)**	7.8	4.7	7.8	4.5	0.95

Data are presented as the mean and standard deviation (SD). P-values are based on a t-test or Wilcoxon rank sum test. Statistically significant (p < 0.05) values are presented in bold. *DOS*: day of surgery; *CRP*: C-reactive protein; *CCl*: creatinine-clearance; *WBC*: white blood cell count; *BUN*: blood urea nitrogen.

**Figure 2:**
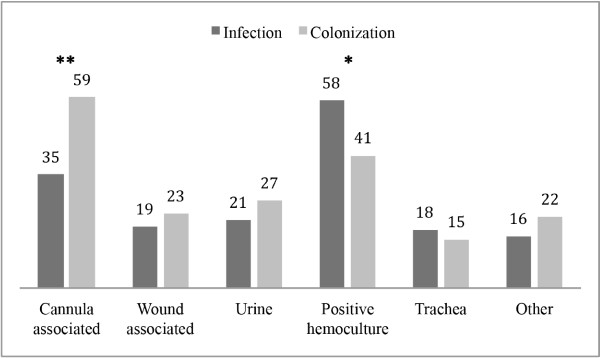
**Positive microbiological results by source.** Data are presented as number of occurrences.

The occurrence of Gram-positive bacteria was significantly greater in the colonization group (p < 0.001) (Table 3). After analyzing the individual pathogens, the occurrence of *coagulase*-*negative Staphylococci* (*CNS*) was significantly greater in the colonization group (p = 0.001), while the presentation of *S*. *aureus* was more frequent in the infection group (p = 0.002) (Table 3). Table 4 and Figure 2 show the sources of the positive microbiological samples in 5 groups (cannula and wound associated, urine, hemoculture and other). The occurrence of the cannula-associated findings was greater in the colonization group, while positive hemo-cultures were significantly more frequent in the infection group (p = 0.004 and p = 0.04, respectively).

**Table 3:** Microbiological results-pathogens

	**After propensity matching**				
	**Infection (n=141)**		**Colonization (n=141)**		
**Variable**	**N**	**%**	**N**	**%**	**p-value**
**Gram negative**	48	34%	50	35%	0.92
**Gram positive**	84	59%	99	70%	**0.04**
**Fungal**	9	6%	7	5%	0.62
**Enterobacter sp.**	10	7%	13	9%	0.39
**Klebsiella**	8	6%	7	5%	0.75
**Pseudomonas sp.**	17	12%	16	11%	0.83
**Coag. Neg. Staph. Aureus**	45	32%	73	52%	**0.001**
**Staph. Aureus**	21	15%	9	6%	**0.002**
**Enterococcus sp.**	11	8%	14	10%	0.42
**Other**	29	20%	31	22%	0.79

Data are presented as the number of occurrences and the percentage. P-values are based on two-sided chi squared or Fischer’s exact tests (as appropriate). Statistically significant (p < 0.05) values are presented in bold.

**Table 4:** Microbiological results-source of positive samples

	**After propensity matching**				
	**Infection (n=141)**		**Colonization (n=141)**		
**Variable**	**N**	**%**	**N**	**%**	**p-value**
**Cannula associated**	35	25%	59	42%	**0.004**
**Wound associated**	19	13%	23	16%	0.58
**Urine**	21	15%	27	19%	0.42
**Positive hemoculture**	58	41%	41	29%	**0.04**
**Trachea**	18	13%	15	11%	0.71
**Other**	16	11%	22	16%	0.27

Data are presented as the number of occurrences and the percentage. P-values are based on two-sided chi squared or Fischer’s exact tests (as appropriate). Statistically significant (p < 0.05) values are presented in bold.

Table 5 shows the summarized outcomes according to the two groups. Before propensity matching, the patients showing signs of infection required renal replacement therapy and reoperations more frequently and had more pulmonary and cardiac complications and longer ICU stays and mechanical ventilation durations. After propensity matching, postoperative nonvascular pulmonary complications were more frequent in the infection group (p = 0.013), the length of the ICU and combined hospital stay and also the mechanical ventilation were longer in the infection group (p= 0.006, p = 0.002 and p = 0.006, respectively).

**Table 5:** Adverse outcomes in the two groups

	**Before propensity matching**					**After propensity matching**				
	**Infection (n = 179)**		**Colonization (n=253)**			**Infection (n=141)**		**Colonization (n=141)**		
**Variable**	**Mean/N**	**SD/%**	**Mean/N**	**SD/%**	**p-value**	**Mean/N**	**SD/%**	**Mean/N**	**SD/%**	**p-value**
**Hospital days**	26.1	12.5	19.7	8.4	<0.001	25.9	13.11	21.3	9.1	**0.002**
**Mortality**	10	6%	19	8%	0.431	7	5%	14	10%	0.06
**Dialysis**	41	23%	27	11%	0.001	23	16%	22	16%	0.31
**Pulmonary**	80	45%	53	21%	<0.001	57	40%	36	26%	**0.01**
**Cardiac**	100	56%	89	35%	<0.001	74	52%	65	46%	0.32
**Cyanosis**	50	28%	63	25%	0.48	43	30%	36	26%	0.41
**Postop. Reoperation**	35	20%	18	7%	<0.001	26	18%	16	11%	0.13
**ICU stay (day)**	10.4	7.56	6.3	5.3	0.001	10	7.42	7.9	6.21	**0.006**
**Extubation time (day)**	213.7	202.4	97.9	149.2	<0.001	184.8	168.3	132	157.1	**0.006**

Data are presented as the number of occurrences and the percentage or as the mean and standard deviation (SD) for categorical and continuous variables, respectively. For categorical variables, the p-values are based on two-sided chi squared or Fischer’s exact tests (as appropriate), comparing the infection patients to the colonization patients. For continuous variables, the p-values are based on a t-test or Wilcoxon rank sum test. Statistically significant (p < 0.05) values are presented in bold. *ICU*: intensive care unit.

## Discussion

We found that postoperative infection occurred relatively frequently (9.3%) in our pediatric cardiac population. These patients had greater postoperative CRP, WBC and BUN values, and also increased occurrence of *S*. *aureus* and positive bloodstream results and more adverse outcomes. Bacterial colonization was significantly more common in this population (15.2%), and after propensity matching analysis, our results showed that the occurrence of *CNS*, Gram-positive bacteria, and positive cannula associated results were greater in this group.

Wound associated infections following cardiac surgery contribute considerably to morbidity and mortality [20, 21]. The estimated SSI risk associated with cardiothoracic procedures is as great as 33% [22]. *S*. *aureus* is the most common pathogen responsible for wound infections, and its colonization of the anterior nares appears to be a major factor in the development of SSIs [23-25]. Delayed sternal closure is associated with not only increased risk of SSI but also an increased occurrence of bloodstream infections [26]. Management strategies, such as preoperative eradication of *S*. *aureus*, are shown to be effective in preventing SSI and controlling *Methicillin*-*resistant S*. *aureus* (*MRSA*) outbreaks [27,28]; however, the continuation of prophylactic antibiotic therapy after delayed sternal closure raises questions regarding the induction of antimicrobial resistance [29,30]. Intranasal mupirocin and perioperative naso- and oropharyngeal application of chlorhexidine have been shown to reduce the rates of deep sternal wound, lower respiratory tract infection and SSIs [31].

The initiation of inflammatory processes during CPB is now widely recognized [32-35]. Although our findings of a relationship between greater CRP, leukocyte count and conversion in the first two days of the postoperative course hints at a possible role of intraoperative initiation of the immune system, these laboratory results have already failed at predicting the occurrence of infection [36]. The association between high CRP and leukocyte levels and infection might be explained as an augmented reaction to CPB and infective agents. There have been attempts at creating simple risk prediction rules, which may be useful for prevention, but because of a lack of widespread validation, there are still major differences in local management strategies [37,38].

After assessing the spectrum of pathogens appearing in our patients, the occurrence of strains capable of entering through and growing on intravenous catheters has evidently been greater in patients lacking any signs of clinical infection, which further indicates the importance of nosocomial transfection of these pathogens. Local catheter treatment solutions, e.g., chlorhexidine-impregnation, have been around for some time but with mixed results [12], while the effectiveness of barrier precaution alone is shown to have a 25% benefit in preventing nosocomial bloodstream infections in the ICU [39].

Death in pediatric sepsis is associated with severe hypovolemia and low cardiac output. Compared to adults, oxygen delivery in children, not oxygen extraction, is the major determinant of oxygen consumption [11]. Complex intensive therapy is needed to stop the vicious circle induced by systemic inflammatory cytokines [12]. Furthermore, the diagnosis of postoperative infection can be difficult because the clinical and laboratory signs of an inflammatory process can be caused not only by infection but also the systemic response activated by CPB or tissue damage [13].

The limitations of this study arise from the issues of heterogeneity of the infection cases. After assessing all types of postoperative infection, we still cannot define which risk factors specifically predict the conversion of colonization in a surgical site or a bloodstream infection. We did not use sepsis severity scoring systems for the prediction of possible conversion, although no system has been validated in this context for pediatric cardiac patients. Further limitations come from the fact that we did not compare the antibiotic therapies in our patients. A large, single-center study usually results in a more homogenous group of patients as far as treatment strategy is concerned, and the specific perioperative management of our hospital may differ from others, although our infection cases were similar to those reported in the literature.

## Conclusions

In summary, we have tried to compare bacterial colonization with clinical infection in this pediatric cardiac population. Our results show that intraoperative stress related to the use of CPB and the patients’ subsequent responses in the early postoperative period plays an important role. Furthermore, we found independent relationships between the conversion of colonization to infection regarding positive *S*. *aureus* and bloodstream results. However, early recognition is crucial because ad-verse outcomes, such as more postoperative pulmonary complications, longer mechanical ventilation and overall hospital stays, were noticeable in the patients with clinically proven infection. The future of treating patients with infection and differentiating them early on from innocuous bacterial colonization rests in the united effort to evaluate and verify the risk factors and microbiological characteristics of the conversion between the two groups.

## Abbreviations

SSI: Surgical site infection; ICU: Intensive care unit; PIRO: Predisposition Infection Response Organ failure; RACHS: Risk adjustment for congenital heart surgery; WBC: White blood cell count; SIRS: Systemic inflammatory response syndrome; CPB: Cardiopulmonary bypass; CRP: C-reactive protein; BUN: blood urea nitrogen; CNS: Coagulase-negative Staphylococcus; MRSA: Methycillin-restant Staphylococcus aureus.

## Competing interests

The authors declare that they have no competing interests.

## Authors’ contributions

ASZ, DJL and ZCS designed the study. ASZ, DJL, ZCS, and TB collected the clinical data. ASZ, DJL, TB analysed and interpreted the data. ASZ and DJL drafted the manuscript. ASZ, ASZa, ES and JG made critical revision of the manuscript for important intellectual content. ASZ, DJL and TB performed the statistical analysis. All authors read and approved the final manuscript.

## Supplementary Material

Additional file 1: Table S1 Demographic and intraoperative variables before propensity matching. Click here for file
