# Supplementary material for: Postoperative differences between colonization and infection after pediatric cardiac surgery-a propensity matched analysis
Source: J Cardiothorac Surg. 2013 Jul 2;8:166. doi: 10.1186/1749-8090-8-166 (PMC3707812; doi:10.1186/1749-8090-8-166)
Supplement: Additional file 1: Table S1 — Demographic and intraoperative variables before propensity matching. [file 1749-8090-8-166-S1.doc]

Supplementary table: Demographic and intraoperative variables before propensity matching

|  | Before propensity matching | | | | |  |
| --- | --- | --- | --- | --- | --- | --- |
|  | Infection | |  | Colonization | |  |
|  | (n = 179) | |  | (n=253) | |  |
| Variable | Mean/N | SD/% |  | Mean/N | SD/% | p-value |
|  |  |  |  |  |  |  |
| **Age (days)** | 396 | 902.7 |  | 691.2 | 1275.9 | **<0.001** |
| **Gender (male)** | 102 | 57% |  | 140 | 55% | 0.73 |
| **Reoperation** | 21 | 12% |  | 31 | 12% | **0.09** |
| **RACHS (point)** | 2.8 | 1.09 |  | 2.6 | 1.06 | 0.52 |
| **Preop. cyanosis** | 80 | 45% |  | 122 | 48% | 0.46 |
| **Preop. ICU** | 87 | 49% |  | 81 | 32% | **<0.001** |
| **Preop. inotrope** | 49 | 27% |  | 39 | 15% | **0.002** |
| **Preop. captopril** | 47 | 26% |  | 47 | 19% | 0.057 |
| **Pulmonary hypertension** | 59 | 33% |  | 52 | 21% | **0.004** |
| **Down's syndrome** | 22 | 12% |  | 10 | 4% | **0.001** |
| **CPB time (min)** | 135.7 | 67.1 |  | 122.5 | 77.4 | 0.18 |
| **Cross-clamp time (min)** | 50.5 | 45.7 |  | 44.3 | 47.5 | 0.46 |
| **Operation time (min)** | 230.2 | 129.4 |  | 211.8 | 124.1 | 0.63 |
| **T min nasal (°C)** | 30.8 | 4.8 |  | 31.8 | 3.86 | **0.01** |
| **DHCA** | 13 | 7% |  | 9 | 4% | 0.08 |
| **Ultrafiltration** | 8 | 4% |  | 9 | 4% | 0.75 |
| **Nitric oxide** | 38 | 21% |  | 32 | 13% | **0.01** |
| **Fluid balance (ml/kg)** | 28.9 | 25.5 |  | 21.3 | 30.4 | 0.83 |
| **RBC (ml/kg)** | 49.7 | 44.2 |  | 32.6 | 34.6 | **0.004** |
| **Aprotinin** | 42 | 23% |  | 71 | 28% | 0.41 |
